# A simple method to assess and report thematic saturation in qualitative research

**DOI:** 10.1371/journal.pone.0232076

**Published:** 2020-05-05

**Authors:** Greg Guest, Emily Namey, Mario Chen

**Affiliations:** 1 Q42 Research, Research Triangle Park, North Carolina, United States of America; 2 Global Health, Population, and Nutrition, FHI 360, Durham, North Carolina, United States of America; University of Birmingham, UNITED KINGDOM

## Abstract

Data saturation is the most commonly employed concept for estimating sample sizes in qualitative research. Over the past 20 years, scholars using both empirical research and mathematical/statistical models have made significant contributions to the question: How many qualitative interviews are enough? This body of work has advanced the evidence base for sample size estimation in qualitative inquiry during the design phase of a study, *prior* to data collection, but it does not provide qualitative researchers with a simple and reliable way to determine the adequacy of sample sizes *during* and/or *after* data collection. Using the principle of saturation as a foundation, we describe and validate a simple-to-apply method for assessing and reporting on saturation in the context of inductive thematic analyses. Following a review of the empirical research on data saturation and sample size estimation in qualitative research, we propose an alternative way to evaluate saturation that overcomes the shortcomings and challenges associated with existing methods identified in our review. Our approach includes three primary elements in its calculation and assessment: Base Size, Run Length, and New Information Threshold. We additionally propose a more flexible approach to reporting saturation. To validate our method, we use a bootstrapping technique on three existing thematically coded qualitative datasets generated from in-depth interviews. Results from this analysis indicate the method we propose to assess and report on saturation is feasible and congruent with findings from earlier studies.

## Introduction

Data saturation is the conceptual yardstick for estimating and assessing qualitative sample sizes. During the past two decades, scholars have conducted empirical research and developed mathematical/statistical models designed to estimate the likely number of qualitative interviews needed to reach saturation for a given study. Although this body of work has advanced the evidence base for sample size estimation during the design phase of a qualitative study, it does not provide a method to determine saturation, and the adequacy of sample sizes, *during* and/or *after* data collection. As Morse pointed out more than 20 years ago, “saturation is an important component of rigor. It is present in all qualitative research but, unfortunately, it is evident mainly by declaration” [1]. In this paper we present a method to assess and report on saturation that enables qualitative researchers to speak about--and provide some evidence for--saturation that goes beyond simple declaration.

To provide the foundation for this approach, we define saturation and then review the work to date on estimating saturation and sample sizes for in-depth interviews. We follow this with an overview of the few empirically-based methods that have been put forward to operationalize and measure saturation and identify challenges of applying these approaches to real-life research contexts, particularly those that use inductive thematic analyses. We subsequently propose an alternative way of evaluating saturation and offer a relatively easy-to-use method of assessing and reporting on it during or after an inductive thematic analysis. We test and validate our method using a bootstrapping technique on three distinctly different qualitative datasets.

The method we propose is designed for qualitative data collection techniques that aim to generate narratives–i.e., focus groups and one-on-one interviews that use open-ended questioning with inductive probing (though we have only attempted to validate the method on individual interview data). Our method also specifically applies to contexts in which an inductive thematic analysis [2–4] is used, where emergent themes are discovered in the data and then transformed into codes.

### A brief history of saturation and qualitative sample size estimation

How many qualitative interviews are enough? Across academic disciplines, and for about the past five decades, the answer to this question has usually revolved around reaching saturation [1, 5–9]. The concept of saturation was first introduced into the field of qualitative research as “theoretical saturation” by Glaser and Strauss in their 1967 book *The Discovery of Grounded Theory* [10]. They defined the term as the point at which “no additional data are being found whereby the [researcher] can develop properties of the category” (pg. 61). Their definition was specifically intended for the practice of building and testing theoretical models using qualitative data and refers to the point at which the theoretical model being developed stabilizes. Many qualitative data analyses, however, do not use the specific grounded theory method, but rather a more general inductive thematic analysis. Over time, the broader term “data saturation” has become increasingly adopted, to reflect a wider application of the term and concept. In this broader sense, saturation is often described as the point in data collection and analysis when new incoming data produces little or no new information to address the research question [4, 9, 11–13].

Interestingly, empirical research on saturation began with efforts to determine when one might *expect* it to be reached. Though “interviewing until saturation” was recognized as a best practice, it was not a sufficient description of sample size. In most research contexts, sample size specification and justification is required by funders, ethics committees, and other reviewers *before* a study is implemented [14, 15]. Applied qualitative researchers faced the question: How do I estimate how many interviews I’ll need before I head into the field?

Empirical research to address this issue began appearing in the literature in the early 2000s. Morgan et al. [16] conducted a pioneer methodological study using data collected on environmental risks. They found that the first five to six interviews produced the majority of new information in the dataset, and that little new information was gained as the sample size approached 20 interviews. Across four datasets, approximately 80% to 92% of all concepts identified within the dataset were noted within the first 10 interviews. Similarly, Guest et al. [9] conducted a stepwise inductive thematic analysis of 60 in-depth interviews among female sex workers in West Africa and discovered that 70% of all 114 identified themes turned up in the first six interviews, and 92% were identified within the first 12 interviews. Subsequent studies by Francis et al. and Namey et al. [17, 18] reported similar findings. Building on these earlier studies, Hagaman and Wutich [19] calculated saturation within a cross-cultural study and found that fewer than 16 interviews were enough to reach data saturation at each of the four sites but that 20–40 interviews were necessary to identify cross-cultural meta-themes across sites.

Using a meta-analytic approach, Galvin [20] reviewed and statistically analyzed—using binomial logic—54 qualitative studies. He found the probability of identifying a concept (theme) among a sample of six individuals is greater than 99% if that concept is shared among 55% of the larger study population. Employing this same logic, Fugard and Potts [21] developed a quantitative tool to estimate sample sizes needed for thematic analyses of qualitative data. Their calculation incorporates: (1) the estimated prevalence of a theme within the population, (2) the number of desired instances of that theme, and (3) the desired power for a study. Their tool estimates, for example, that to have 80% power to detect two instances of a theme with a 10% prevalence in a population, 29 participants would be required. Note that their model assumes a random sample.

The above studies are foundational in the field of qualitative sample size estimation. They provide empirically-based guidance for approximating how many qualitative interviews might be needed for a given study and serve a role analogous to power calculations in quantitative research design (albeit in some case without the math and degree of precision). And, like power calculations, they are moot once data collection begins. Estimates are based on (specified) assumptions, and expectations regarding various elements in a particular study. As all researchers know, reality often presents surprises. Though a study may be powered to certain parameters (quantitative) or have a sample size based on empirical guidance (qualitative), after data collection is completed the resulting data may not conform to either.

Not surprisingly, researchers have recently begun asking two follow up questions about data saturation that go beyond estimation: *How can we better operationalize the concept of saturation*? and *How do we know if we have* *reached* *saturation*?

### Operationalizing and assessing saturation

The range of empirical work on saturation in qualitative research and detail on the operationalization and assessment metrics used in data-driven studies that address saturation are summarized in Table 1. In reviewing these studies to inform the development of our approach to assessing saturation, we identified three limitations to the broad application of saturation assessment processes which we sought to overcome: lack of comparability of metrics, reliance on probability theory or random sampling, and retrospective assessment dependent on having a fully coded/analyzed dataset. We discuss each limitation briefly before introducing our alternative approach.

**Table 1:** Summary of data-driven saturation studies.

Study Authors	Approach/Process	Characteristics
Guest et al. [9]	Operationalize saturation as a proportion: the number of identified themes at a given point in analysis divided by the total number of themes identified in the entire sample. Level of saturation reported as the point at which, *post facto*, 80% or 90% of themes in a dataset are identified. Findings additionally validated by determining the point during analysis when the most prevalent themes were identified.	• Conducted saturation analysis in batches of 6 interviews • Findings contributed to estimating sample sizes when designing a study • Because the denominator (number of interviews in a dataset) is fixed while the numerator gets closer to the denominator with every new interview, all datasets will eventually reach 100% saturation
Francis et al. [17]	Authors posit that researcher should establish two key parameters prior to analysis: *Initial Analysis Sample*–the prespecified number of interviews included in the first round of data analysis. *Stopping Criterion*–the number of consecutive interviews, after the initial sample is analyzed, in which no new themes are identified. Saturation achieved if no new themes are identified in *x* number of consecutive interviews past the initial analysis sample (stopping criterion).	• Saturation requirements are strict and difficult to achieve in smaller studies • Specified for theory-based studies • Only one initial sample size and stopping criterion were tested.
Coenen et al. [22]	Operationalize saturation as the point at which linking concepts from two consecutive unique interviews reveals no additional second-level categories.	• Saturation depends on the relationship between only two interviews • Determining saturation is vulnerable to outliers • Definition and inclusion of second-level categories is unclear
Galvin [20]	Meta-analysis of 54 published studies in the Building & Energy research literature. Employed a statistical approach, based on binomial logic, to ascertain the relationship between theme identification in a particular sample and the larger population (e.g., *n*% chance of detecting a theme, if that theme exists within *n*% of the population).	• Probability-based • Assumes a random sample • Assumes it is possible to know in advance what particular (emergent) themes will be and at what rate they might occur; not suitable for inductive studies
Fugard & Potts [21]	Statistical calculation of saturation that includes: a) Expected theme prevalence within the population (derived from either prior knowledge, or the prevalence of the rarest themes of interest) b) Number of desired instances of the theme c) Desired power of study	• Based on probability theory • Requires knowing and specifying several parameters prior to data collection • Assumes it’s possible to know in advance what particular (emergent) themes will be and at what rate they might occur; not suitable for inductive studies
Hennink et al. [23]	Distinguish between “code” and “meaning” saturation. Code saturation calculation includes primary/parent codes. Meaning saturation calculation also includes more nuanced sub/child codes. Saturation operationalized as the proportion of identified codes or code details (“meaning”) at a given point in analysis divided by the total number identified in the entire sample. Findings additionally validated by determining when most prevalent codes identified during analysis.	• Findings contributed to estimating sample sizes when designing a study • Because the denominator (number of interviews in a dataset) is fixed while the numerator gets closer to the denominator with every new interview, all datasets will eventually reach 100% saturation
Tran et al. [24]	Used Monte Carlo simulation on open-ended survey questions to predict the number of themes discovered with the inclusion of new participants. Authors then tested how the slope of the expected theme accumulation curve could be used to determine a stopping criterion for data collection (i.e., saturation). Saturation defined as the point in data collection at which the cost of including a new unit of analysis exceeds the expected gain in information.	• Based on open-ended responses within a survey • Not generalizable to free-flowing text, since parameters of the responses are more constrained than in a conversational style interview
Rijnsoever [25]	Uses mathematical simulations to compare three different research context scenarios in their ability to demonstrate data saturation in a sample relative to the study population. Saturation is “reached after all the codes in the population have been observed once in the sample”.	• Population ‘data’ is hypothetical • Simulation model requires large amounts of *a priori* information
Hagaman & Wutich [19]	Authors first identified the three most prevalent unique themes in a dataset. Used a bootstrapping technique to randomly order interview data. Documented the average number of interviews required to observe the first, second, and third occurrence of the three most prevalent themes in each dataset.	• Provides estimates for cross-site samples • Must first identify most prevalent unique themes
Lowe et al. [26]	Saturation measure based on mathematical representation of the rate of theme discovery during a research process. Compared four different statistically-based models, each with different assumptions about the degree of assumed dependency between observations. Models validated on two different datasets–one derived from focus groups, the other from literature surveys. Models were compared in their ability to estimate sample size before and during data collection.	• Probability-based; assumes a random sample • Equations are complex and not accessible to many qualitative researchers
Weller et al. [27]	Test a refined definition of “saturation” based on the *most salient* items in sets of free-lists generated through interviews. General linear models employed on 28 free-listing datasets to predict unique number of items added by each interview respondent. Saturation defined as point where the expected number of new items was one or less.	• Datasets from free-listing activities • “Salience” measure not applicable to more traditional forms of in-depth interviews or datasets composed of free-flowing narrative

#### Lack of comparability in metrics

Current operationalizations of saturation vary widely in the criteria used to arrive at a binary determination of saturation having been reached or not reached (e.g., Francis et al. [17] and Coenen et al. [22]). Given how different approaches are–in terms of units of analysis and strictness of saturation thresholds–it is difficult to understand how much confidence to have in a conclusion about whether saturation was reached or not. Unlike quantitative researchers using statistical analysis methods who have established options for levels of confidence intervals and other metrics to report, there are no agreed-upon metrics to help qualitative researchers interpret the strength of their saturation findings. The method we propose facilitates qualitative researchers’ choice among levels of assessment criteria along with a common description of those criteria that will allow readers to interpret conclusions regarding saturation with more or less confidence, depending on the strictness of the criteria used.

#### Reliance on probability theory, and/or the assumption of a random sample

Basing assessments of saturation on probabilistic assumptions (e.g., Lowe et al. [26], Fugard & Potts [21], Galvin [20]) ignores the fact that most qualitative research employs non-probabilistic, purposive sampling suited to the nature and objectives of qualitative inquiry [28]. Even in cases where random sampling is employed, the open-ended nature of qualitative inquiry doesn’t lend itself well to probability theory or statistical inference to a larger population because response categories are not structured, so are not mutually exclusive. The expression of Theme A is not necessarily to the exclusion of Theme B, nor does the absence of the expression of Theme A necessarily indicate Not-A. Further, from a logistical standpoint, many qualitative researchers do not have the expertise, nor the time required, to perform complicated statistical tests on their datasets. Our approach involves only simple arithmetic and calculation of percentages.

#### Retrospective assessment dependent on having a fully coded/analyzed dataset

Methods that calculate saturation based on the proportion of new themes relative to the overall number of themes in a dataset (e.g., Guest et al. [9], Hennink et al. [23]) are limited by the total number of interviews conducted: the denominator represents the total number of themes in the fully-analyzed dataset and is fixed, while the number of themes in the numerator gets closer to the denominator with every new interview considered, thus eventually reaching 100% saturation. Saturation will inevitably occur in a retrospectively-assessed, fully-analyzed, fixed-size dataset. The method we outline eliminates this problem by using a subset of data items in the denominator instead of the entire dataset, facilitating better prospective assessment of saturation and offering the advantage of allowing researchers to stop before reaching a pre-specified number of interviews. (Under our approach, however, a measure of percent saturation as defined by these authors will not be available.)

## Methods

### An alternative approach and method to calculating and reporting saturation

For the purposes of our assessment, saturation refers to the point during data analysis at which incoming data points (interviews) produce little or no new useful information relative to the study objectives. Our approach to operationalizing this definition of saturation consists of three distinct elements–the *base size*, the *run length*, and the relative amount of incoming new information, or the *new information threshold*.

#### Base size

When assessing saturation, incoming information is weighed against the information already obtained. **Base size** refers to how we circumscribe the body of information already identified in a dataset to subsequently use as a denominator (similar to Francis et al.’s initial analysis sample). In other words, what is the minimum number of data collection events (i.e., interviews) we should review/analyze to calculate the *amount of information already gained*? We know that if we use all of the data collection events as our base size, we can reach saturation by default as there are no more data to consider. We also know from previous studies [9, 16, 29] that most novel information in a qualitative dataset is generated early in the process, and generally follows an asymptotic curve, with a relatively sharp decline in new information occurring after just a small number of data collection/analysis events. For this reason, we have chosen to test 4, 5, and 6 interviews as base sizes from which to calculate the total number of unique themes to be used in the denominator of the saturation ratio. The unit of analysis for base size is the data collection event; the items of analysis are unique codes representing themes.

#### Run length

**Fig 1:**
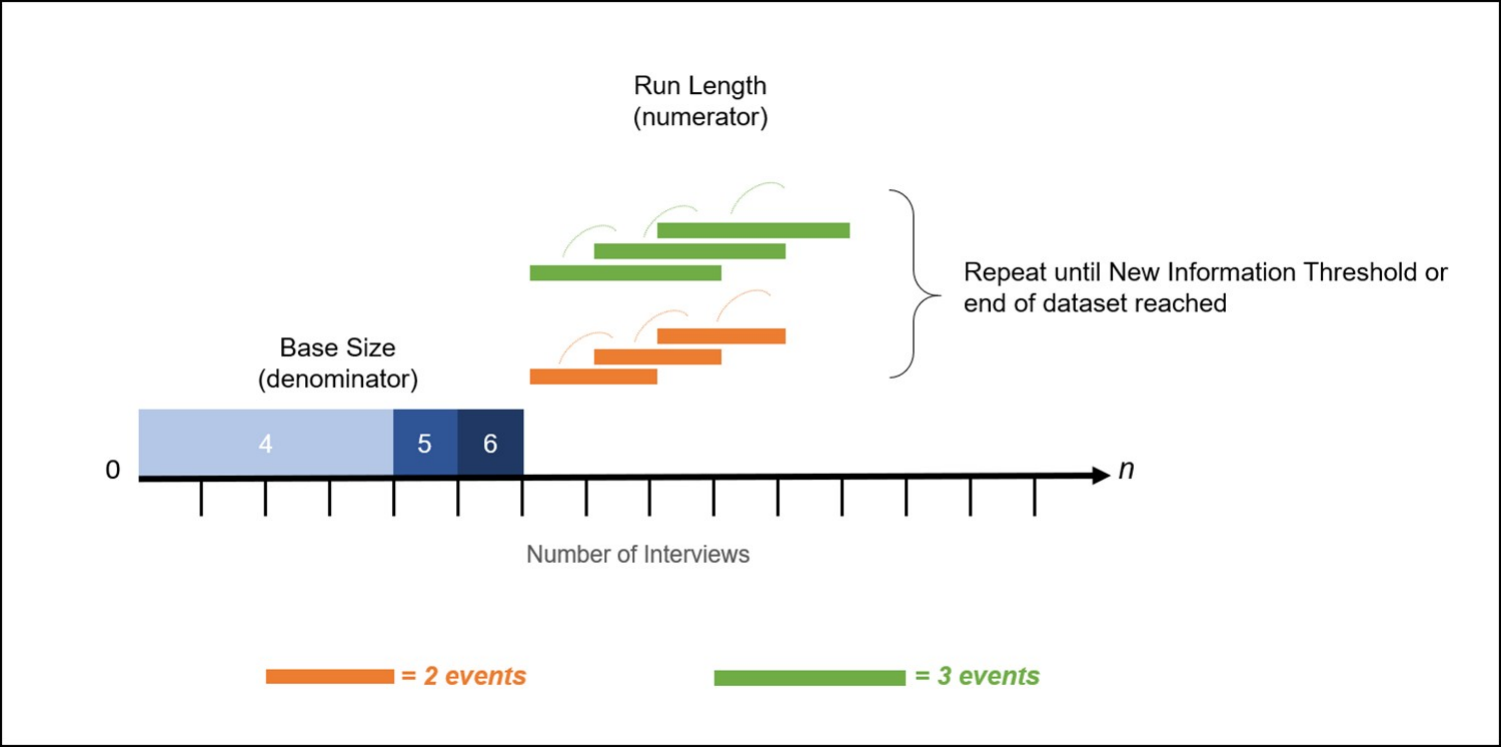
Summary of process, base size and run length options.

A run can be defined as a set of consecutive events or observations, in this case interviews. The **run length** is the *number of interviews* within which we look for, and calculate, *new information*. The number of new themes found in the run defines the numerator in the saturation ratio. Hagaman and Wutich (2017) and Francis et al. (2010), for example, consider runs of three data collection events each time they (re)assess the number of new themes for the numerator, whereas Coenen et al. (2012) include only two events in their data runs. For our analyses we provide both options for run lengths in our calculations–two events and three events–to afford researchers more flexibility. Note that in our analyses, successive runs overlap: each set of interviews shifts to the right or “forward” in time by one event. Fig 1 shows the process, and how base size and run length relate to one another. Here again the unit of analysis is the data collection event; the items of analysis are unique codes.

#### New information threshold

Once units of analysis for the numerator and denominator are determined the proportional calculation is simple. But the next question is a purely subjective one: What level of paucity of new information should we accept as indicative of saturation? We propose that furnishing researchers with options—rather than a prescriptive threshold—is a more realistic, transparent and accurate practice. We therefore propose initially two levels of new information that represent the proportion of new information we would accept as evidence that saturation has been reached at a given point in data collection: ≤5% new information and no (0%) new information.

**Fig 2:**
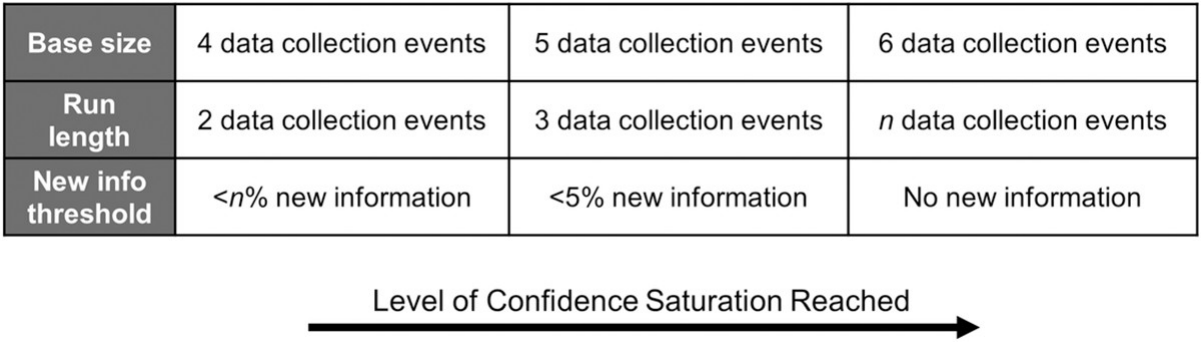
Saturation assessment parameters and level of confidence saturation reached.

These new information thresholds can be used as benchmarks similar to how a p-value of <0.05 or <0.01 is used to determine whether enough evidence exists to reject a null hypothesis in statistical analysis. As in statistical analysis—but absent the probability theory—there is no guarantee that saturation is in fact reached when meeting these thresholds. But they do provide a transparent way of presenting data saturation assessments that can be subsequently interpreted by other researchers. The lower the new information threshold, the less likely an important number of themes may remain undiscovered in later interviews if data collection stops when the threshold is reached. Taken together, the concepts of base size, run length, and new information threshold allow researchers to choose how stringently they wish to apply the saturation concept–and the level of confidence they might have that data saturation was attained for a given sample (Fig 2).

The advantages of the method we propose are several:

- It does not assume or require a random sample, nor prior knowledge of theme prevalence.
- Calculation is simple. It can be done quickly and with no statistical expertise.
- Metrics can be used *prospectively* during the data collection and analysis process to ascertain when saturation is reached (and providing the possibility of conducting fewer data collection events than planned).
- Metrics can be used *retrospectively*, after data collection and analysis are complete, to report on the adequacy of the sample to reach thematic saturation.
- Options for each metric can be specified prior to analysis or reported after data analysis.
- The metrics are flexible. Researchers have options for how they describe saturation and can also use the term with more transparency and precision.
- Saturation is conceptualized as a relative measure. This neutralizes differences in the level of coding granularity among researchers, as the method affects both numerator and denominator.

### Application of the approach

#### An example of prospective data saturation calculation

**Fig 3:**
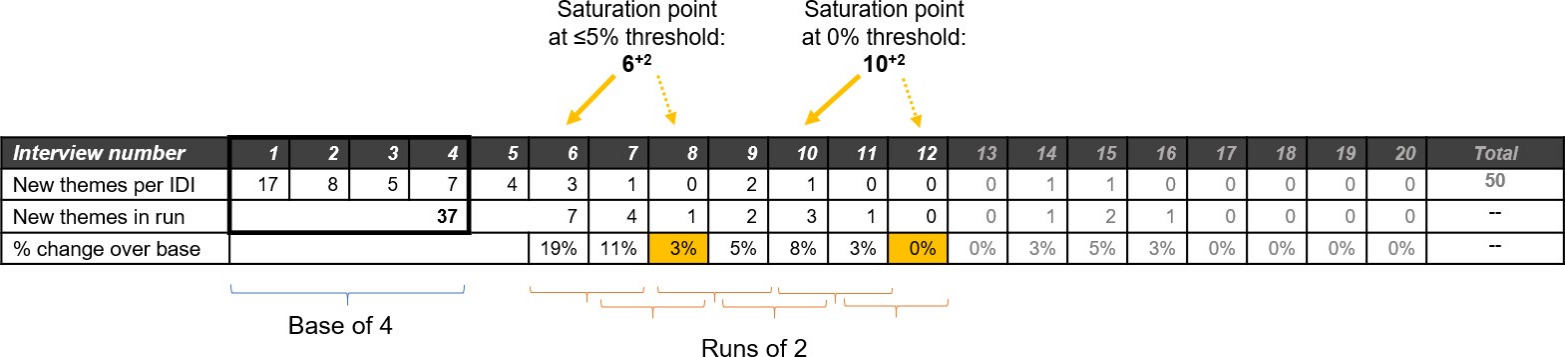
Hypothetical data for example of saturation assessment at base size 4 and run length 2.

Let’s consider a step-by-step example of how this process works, using a hypothetical dataset to illustrate the approach. We will prospectively calculate saturation using a **base size of 4** interviews and **run length of 2** interviews. For this example, we have selected a **new information threshold of** ≤**5%** to indicate that we have reached adequate saturation. [The data used for each step are included in Fig 3, along with indication of the base, runs, and saturation points.]

#### STEP 1 –Find the number of unique themes for base

We start by looking at the first four interviews conducted and summing the number of unique themes identified within this group. The resulting sum, 37, is the denominator in our equation.

**Table:** 

*Interview number*	*1*	*2*	*3*	*4*
New themes per interview	17	8	5	7
# Base themes				**37**

#### STEP 2—Find the number of unique themes for the first run

In this example, we’re using a run length of two, so include data for the next two interviews after the base set–i.e., interviews 5 and 6. After reviewing those interviews, let’s say we identified four new themes in interview 5 and three new themes in interview 6. The number of new themes in this first run is seven.

**Table:** 

*Interview number*	*5*	*6*
New themes per interview	4	3
New themes in run		**7**

#### STEP 3 –Calculate the saturation ratio

Divide the number of new themes in this run (seven) by the number of unique themes in the base set (37). The quotient reveals 19% new information. This is not below our ≤5% threshold, so we continue.

**Table:** 

# New themes/run	=	7	=	**19%**
# Base themes		37		

#### STEP 4 –Find the number of new unique themes for the next run in the series

For the next run we add the new themes for the next two interviews, 6 and 7 (note the overlap of interview 6), resulting in a sum of four.

**Table:** 

*Interview number*	*6*	*7*
New themes per interview	3	1
New themes in run		**4**

#### STEP 5—Update saturation ratio

Take the number of new themes in the latest run (four) and divide by the number of themes in the base set (37). This renders a quotient of 11%, still not below our ≤5% threshold. We continue to the next run.

**Table:** 

# New themes/run	=	4	=	**11%**
# Base themes		37		

#### STEP 6 –Find the number of new unique themes for the next run in the series

For this third run we add the number of new themes identified within interviews 7 and 8.

**Table:** 

*Interview number*	*7*	*8*
New themes per interview	1	0
New themes in run		**1**

#### STEP 7—Update saturation ratio

Take the number of new themes in the latest run (one) divided by the number of themes in the base set (37).

**Table:** 

# New themes/run	=	1	=	**3%**
# Base themes		37		

At this point the proportion of new information added by the last run is below the ≤5% threshold we established, so we stop here after the 8^th^ interview and have a good sense that the amount of new information is diminishing to a level where we could say saturation has been reached based on our subjective metric of ≤5%. Since the last two interviews did not add substantially to the body of information collected, we would say that saturation was reached at interview 6 (each of the next two interviews were completed to see how much new information would be generated and whether this would fall below the set threshold). We would annotate these two extra interviews (indicative of run length) by appending a superscript “+2” to the interview number, to indicate a total of eight interviews were completed. In writing up our saturation assessment then, we would say that using a base size 4 we reached the ≤5% new information threshold at 6^+2^ interviews.

If we wanted to be more conservative, and confident in our conclusion of reaching saturation in this example, we could adjust two parameters of our assessment. We could increase the run length to 3 (or an even larger number), and/or we could set a more stringent new information threshold of no new information. If we consider the hypothetical data set used here (see Fig 3) and kept the run length of 2, the 0% new information threshold would have been reached at interview 10^+2^.

One may still raise two logical questions after reviewing the example process above. The first is “How do we know that we’re not missing important information by capping our sample at *n* when saturation is indicated?” Put another way, if we had conducted, say, five more interviews would we have gotten additional and important data? The honest answer to this is that we don’t know, and we can never know unless we conduct those five extra interviews, and then five more after that and so on. That is where we rely on the empirical research that shows the rate at which new information emerges decreases over time and that the most common and salient themes are generated early, assuming that we keep the interview questions, sample characteristics, and other study parameters relatively consistent. To further illustrate how saturation may have been affected by doing additional interviews, we include 20 interviews in Fig 3. The interviews following Interview 12, though yielding four additional themes, remained at or below the ≤5% new information threshold.

The second question is to a degree related to the first question and pertains to possible order effects. Would the theme identification pattern in a dataset of 20 interviews look the same if interviews #10 through #20 were conducted first? Could new themes start emerging later in the data collection process? Though it is possible an important theme will emerge later in the process/dataset, the empirical studies referenced above demonstrate that the most prevalent, high-level, themes are identified very early on in data collection, within about six interviews. But, to further check this, we use a bootstrapping technique on three actual datasets to corroborate findings from these earlier studies and to assess the distributional properties of our proposed metrics. These bootstrap findings give us information on how saturation may be reached at different stopping points as new themes are discovered in new interviews and when the interviews are ordered randomly in different replications of the sample of interviews.

#### Sample datasets

We selected three existing qualitative datasets to which we applied the bootstrapping method. Although the datasets were all generated from individual interviews analyzed using an inductive thematic analysis approach, the studies from which they were drawn differed with respect to study population, topics of inquiry, sample heterogeneity, interviewer, and structure of data collection instrument, as described below.

*Dataset 1*. This study included 40 individual interviews with African American men in the Southeast US about their health seeking behaviors [29]. The interview guide contained 13 main questions, each with scripted sub-questions. Inductive probing was employed throughout all interviews. The inductive thematic analysis included 11 of the 13 questions and generated 93 unique codes. The study sample was highly homogenous.

*Dataset 2*. The second dataset consists of 48 individual interviews conducted with (mostly white) mothers in the Southeast US about medical risk and research during pregnancy [30]. The interview guide contained 13 main questions, each with scripted sub-questions. Inductive probing was employed throughout all interviews. Of note, the 48 interviews were conducted, 12 each, using different modes of data collection: in-person, by video (Skype-like platform), email (asynchronous), or text chat (synchronous). The qualitative thematic analysis included 10 of these questions and generated 85 unique codes.

*Dataset 3*. This study included 60 interviews with women at higher risk of HIV acquisition—30 participants in Kenya and 30 in South Africa [31]. The interview was a follow-up qualitative inquiry into women’s responses on a quantitative survey. Though there were 14 questions on the guide, only data from three questions were included in the thematic analysis referenced here. Those three questions generated 55 codes. Participants from the two sites were similar demographically with the exceptions of education and marital status. Substantially more women from the Kenya sample were married and living with their partners (63% versus 3%) and were less likely to have completed at least some secondary education. All interviews were conducted in a local language.

Data from all three studies were digitally recorded and transcribed using a transcription protocol [32]; transcripts were translated to English for Dataset 3. Transcripts were imported into NVivo [33] to facilitate coding and analysis. All three datasets were analyzed using a systematic inductive thematic approach [2], and all codes were explicitly defined in a codebook following a standard template [34]. For Datasets 1 & 2, two analysts coded each transcript independently and compared code application after each transcript. Discrepancies in code application were resolved through discussion, resulting in consensus-coded documents. For Dataset 3, two coders conducted this type of inter-coder reliability assessment on 20% of the interviews (a standard, more efficient approach than double-coding all interviews [2]). All three studies were reviewed and approved by the FHI 360 Protection of Human Subjects Committee; the study which produced Dataset 3 was also reviewed and approved by local IRBs in Kenya and South Africa.

#### Bootstrapping method

While these three studies offer diverse and analytically rigorous case studies, they provide limited generalizability. To approximate population-level statistics and broaden our validation exercise, we drew empirical bootstrap samples from each of the datasets described above. The bootstrap method is a resampling technique that uses the variability within a sample to estimate the sampling distribution of metrics (in this case saturation metrics) empirically [35]. This is done by randomly resampling from the sample with replacement (i.e., an item may be selected more than once in a resample) many times in a way that mimics the original sampling scheme. For each qualitative dataset, we generated 10,000 resamples from the original sample. In addition, we randomly ordered the selected transcripts in each resample to offset any order effect on how/when new codes are discovered. For each resample, we calculated the proportion of new themes found in run lengths of two or three new events relative to a base size of four, five or six interviews. We then identified the number of transcripts needed to meet a new information threshold of ≤5% or 0%. Based on these thresholds from 10,000 resamples, for each dataset we computed the median and the 5th and 95th percentiles for number of interviews required to reach each new information threshold across different base sizes and run lengths. The 5th and 95th percentiles provide a nonparametric 90% confidence interval for the number of transcripts needed to reach saturation as defined at these new information thresholds.

Since we had available the total number of codes identified in each dataset, we carried out one additional calculation as a way to provide another metric to understand how the median number of interviews to reach a new information threshold related to retrospectively-assessed degrees of saturation with the entire dataset. In this case, once the number of interviews to reach a new information threshold was determined for each run of a dataset, we divided the number of unique themes identified up to that point by the total number of unique themes. This provided a percent–or degree–of saturation for each run of the data, which was then used to generate a median and 5^th^ and 95^th^ percentile for the **degree of saturation** reached. This can then be compared across base sizes, run lengths, and new information thresholds. [Note that we include this as a further way to understand and validate the proposed approach for calculating saturation, rather than as part of the proposed process.]

## Results

The results from the bootstrapping analyses are presented by dataset, in Tables 2, 3 and 4. Each table presents median and percentiles of the bootstrap distribution using bases of 4, 5 or 6 and run lengths of 2 and 3, at new information thresholds of ≤5% and no new information.

**Table 2:** Dataset 1 bootstrap distribution: Median number of interviews to reach saturation and degree of saturation at different base sizes and run lengths.

Dataset 1 (N = 40 interviews, 93 total unique codes)			Base Size (# of Interviews)					
			4		5		6	
			Run length		Run length		Run length	
New Info Threshold	Saturation Metric		2 IDIs	3 IDIs	2 IDIs	3 IDIs	2 IDIs	3 IDIs
≤5%	Number of IDIs	Median (5^th^ & 95^th^ %ile)	6 (4, 9)	7 (4, 12)	6 (4, 9)	7 (4, 11)	6 (4, 9)	7 (4, 11)
	Degree of Saturation	At Median (5^th^ & 95^th^ %ile)	78% (70%, 87%)	83% (73%, 89%)	78% (70%, 87%)	82% (73%, 89%)	78% (70%, 86%)	82% (73%, 89%)
0%	Number of IDIs	Median (5^th^ & 95^th^ %ile)	11 (5, 17)	14 (7, 23)	11 (5, 17)	14 (7, 23)	11 (5, 17)	14 (7, 23)
	Degree of Saturation	At Median (5^th^ & 95^th^ %ile)	87% (76%, 95%)	90% (82%, 97%)	87% (76%, 95%)	90% (82%, 97%)	87% (76%, 95%)	90% (82%, 97%)

**Table 3:** Dataset 2 bootstrap distribution: Median number of interviews to reach saturation and degree of saturation at different base sizes and run lengths.

Dataset 2 (N = 48 interviews, 85 total unique codes)			Base Size (# of Interviews)					
			4		5		6	
			Run length		Run length		Run length	
New Info Threshold	Saturation Metric		2 IDIs	3 IDIs	2 IDIs	3 IDIs	2 IDIs	3 IDIs
≤5%	Number of IDIs	Median `(5^th^ & 95^th^ %ile)	6 (4, 10)	8 (4, 13)	6 (4, 10)	7 (4, 12)	6 (4, 9)	7 (4, 12)
	Degree of Saturation	At Median (5^th^ & 95^th^ %ile)	79% (69%, 87%)	82% (73%, 89%)	79% (68%, 87%)	82% (73%, 89%)	79% (67%, 87%)	82% (72%, 89%)
0%	Number of IDIs	Median (5^th^ & 95^th^ %ile)	11 (6, 17)	14 (8, 22)	11 (6, 17)	14 (8, 22)	11 (6, 17)	14 (8, 22)
	Degree of Saturation	At Median (5^th^ & 95^th^ %ile)	87% (75%, 93%)	89% (81%, 95%)	87% (75%, 93%)	89% (81%, 95%)	87% (75%, 93%)	89% (81%, 95%)

**Table 4:** Dataset 3 bootstrap distribution: Median number of interviews to reach saturation and degree of saturation at different base sizes and run lengths.

Dataset 3 (N = 60 interviews, 55 total unique codes)			Base Size (# of Interviews)					
			4		5		6	
			Run length		Run length		Run length	
New Info Threshold	Saturation Metric		2 IDIs	3 IDIs	2 IDIs	3 IDIs	2 IDIs	3 IDIs
≤5%	Number of IDIs	Median (5^th^ & 95^th^ %ile)	9 (4, 16)	12 (6, 22)	8 (4, 14)	11 (6, 19)	8 (4, 13)	11 (6, 18)
	Degree of Saturation	At Median (5^th^ & 95^th^ %ile)	64% (45%, 78%)	71% (53%, 84%)	62% (44%, 76%)	69% (53%, 82%)	62% (44%, 76%)	69% (53%, 82%)
0%	Number of IDIs	Median (5^th^ & 95^th^ %ile)	12 (6, 19)	16 (8, 26)	12 (6, 19)	16 (8, 26)	12 (6, 19)	16 (8, 26)
	Degree of Saturation	At Median (5^th^ & 95^th^ %ile)	69% (49%, 83%)	76% (60%, 87%)	69% (49%, 83%)	76% (60%, 87%)	69% (49%, 83%)	76% (60%, 87%)

Note that, as described in the example above, the number of interviews in the run length is not included in the number of interviews to reach the given new information threshold, so the total number of events needed to assess having reached the threshold is two or three *more* interviews than the given median, depending on the run length of choice. This is indicated by a superscript +2 or +3.

For Dataset 1 (Table 2), at the ≤5% new information threshold, the median number of interviews needed to reach a drop-off in new information was consistent across all base sizes. At a run length of two interviews, the median number of interviews required before a drop in new information was observed was six. This means that relative to the total number of unique codes identified in the first four, five, or six interviews, the amount of new information contributed by interviews 7 and 8 was less than or equal to 5% of the total. At a run length of three interviews, the median number of interviews required before a drop in new information was observed was seven. This means that relative to the total number of unique codes identified in the first four, five, or six interviews, the amount of new information contributed by interviews 8, 9, and 10 was less than or equal to 5% of the total. Across base sizes, for a run length of two, we would say that saturation was indicated at 6^+2^, while for a run length of three we would say saturation was observed at 7^+3^, both at the ≤5% new information level. Using the total number of themes in the dataset retrospectively, the number of themes evident across 6–7 interviews corresponded with a median degree of saturation of 78% to 82%.

At the 0% new information threshold, the median number of interviews to indicate saturation were again consistent across bases sizes, varying only by the run length. The median number of interviews required were 11^+2^ and 14^+3^. In other words, at run length 2, it took 11 interviews, plus two more to confirm that no new information was contributed. At run length 3 it was 14 interviews plus three more to confirm no new information. The number of themes evident across 11–14 interviews corresponded with a median degree of saturation of 87% to 89%.

The results for Dataset 2 were nearly identical to Dataset 1 (Table 3). Saturation was indicated at 6 interviews at a run length of 2 (6^+2^) and 7–8 interviews at run length 3 (7^+3^ or 8^+3^). The number of themes evident across 6–8 interviews corresponded with a median degree of saturation of 79% to 82%. At the 0% new information threshold saturation was indicated at the same points as in Dataset 1: 11^+2^ and 14^+3^, consistent across all base sizes. In other words, no new information was observed after a median of 11 interviews using a run-length of 2, nor after 14 interviews using a run length of 3. Here again, despite a different total number of themes in the overall dataset, the number of new themes evident across 11–14 interviews corresponded with a median degree of saturation of 87% to 89%.

Dataset 3 (Table 4) contained more variation in the sample than the others, which was reflected in a slightly higher median number of interviews and a lower degree of saturation. At the ≤5% new information threshold, the median number of interviews required to reach saturation at a run length of 2 was 8–9 (higher for base size 4). At a run length of 3, the median number of required interviews was 11–12 (again higher for base size 4). The number of new themes evident across 8–12 interviews corresponded with a median degree of saturation of 62% to 71%. At the 0% new information threshold, saturation was indicated at 12^+2^ and 16^+3^, consistent across base sizes. The number of new themes evident across 12–16 interviews corresponded with a median degree of saturation of 69% to 76%.

## Discussion

In this paper we present a way of assessing thematic saturation in inductive analysis of qualitative interviews. We describe how this method circumvents many of the limitations associated with other ways of conceptualizing, assessing and reporting on saturation within an in-depth interview context. The process can be applied either *prospectively* during the data collection and analysis process or *retrospectively*, after data collection and analysis are complete. A key advantage is that the metrics are flexible, affording researchers the ability to choose different degrees of rigor by selecting different run lengths and/or new information thresholds. Similarly, the method allows for different options–and greater clarity and transparency–in describing and reporting on saturation.

Based on the bootstrapping analyses we can draw several conclusions. The first is that the results are within the range of what we would have expected based on previous empirical studies. Using the ≤5% new information threshold, our findings indicate that typically 6–7 interviews will capture the majority of themes in a homogenous sample (6 interviews to reach 80% saturation). Our analyses also show that at the higher end of the range for this option (95^th^%ile) 11–12 interviews might be needed, tracking with existing literature indicating 12 interviews are typically needed to reach higher degrees of saturation.

We can also draw other lessons to inform application of this process:

- Base size appears to have almost no effect on the outcome. This is important from an efficiency perspective. If our findings hold true in other contexts, it suggests that using a default base size of four interviews is sufficient. In practical terms, this implies that saturation should initially be assessed after six interviews (four in the base, and two in the run). If analyzing data in real time, the results of this initial assessment can then determine whether or not more interviews are needed.
- Run length has an effect on the outcome, as one would expect. The longer the run length, the greater number of interviews required to reach saturation. The size of run length effect is smallest–very minimal–if employing the ≤5% new information threshold. The practical implication of this finding is that researchers can choose a longer run length–e.g., three interviews (or more)–to generate a more conservative assessment of saturation.
- The new information threshold selected affects the point at which saturation is indicated, as one would expect. The lower the new information threshold–and therefore the more conservative the allowance for recognizing new information–the more interviews are needed to achieve saturation. From an applied standpoint this finding is important in that researchers can feel confident that choosing a more stringent new information threshold–e.g., 0%—will result in a more conservative assessment of saturation, if so desired.

There are, of course, still limitations to this approach. It was developed with applied inductive thematic analyses in mind–those for which the research is designed to answer a relatively narrow question about a specific real-world issue or problem–and the datasets used in the bootstrapping analyses were generated and analyzed within this framework. The applicability of this approach for qualitative research with a different epistemological or phenomenological perspective is yet untested. Another potential limitation of this method relates to codebook structure. When conducting an inductive thematic analysis, researchers must decide on an appropriate codebook organizational scheme (see Hennink et al. [23] for discussion on this as it relates to saturation). We tested our method on single-tier codebooks, but qualitative researchers often create hierarchical codebooks. A two-tier structure with primary (“parent”) codes and constituent secondary (“child”) codes is a common form, but researchers may also want to identify and look for higher-level, meta-themes (e.g., Hagaman and Wutich [19]). For any method of assessing saturation, including ours, researchers need to decide at which level they will identify and include themes/codes. For inductive thematic analyses this is a subjective decision that depends on the degree of coding granularity necessary for a particular analytic objective, and how the research team wants to discuss saturation when reporting study findings. That said, a researcher could, with this approach, run and report on saturation analyses of two or more codebooks that contain differing levels of coding granularity.

## Conclusion

Tran and colleagues [24] accurately point out that determining the point of saturation is a difficult endeavor, because “researchers have information on only what they have found” (pg. 17). They further argue that the stopping point for an inductive study is typically determined by the “judgement and experience of researchers”. We acknowledge and agree with these assertions.

Selecting and interpreting levels of rigor, precision, and confidence is a subjective enterprise. What a quantitative researcher accepts, for example, as a large enough effect size or a small enough p-value is a subjective determination and based on convention in a particular field of study. The same can be said for how a researcher chooses to report and interpret statistical findings. P-values can be expressed either in absolute terms (e.g., *p* = .043) or in several commonly used increments (e.g., *p* < .05, *p* < .01, etc.). Likewise, while an odds ratio of 1.2 may be statistically significant, whether or not it’s meaningful in a real-world sense is entirely open to interpretation.

We are advocating for similar flexibility and transparency in assessing and reporting on thematic saturation. We have provided researchers with a method to easily calculate saturation during or after data collection. This method also enables researchers to select different levels of the constituent elements in the process–i.e., Base Size, Run Length and New Information Threshold–based on how confident they wish to be that their interpretations and conclusions are based on a dataset that reached thematic saturation. We hope researchers find this method useful, and that others build on our work by empirically testing the method on different types of datasets drawn from diverse study populations and contexts.

## Supporting information

S1 Datasets Datasets used in bootstrapping analyses. (XLSX)
